# A 14-year longitudinal study of neurofilament light chain dynamics in premanifest and transitional Huntington’s disease

**DOI:** 10.1007/s00415-024-12700-x

**Published:** 2024-10-03

**Authors:** Z. J. Voysey, N. E. Owen, J. A. Holbrook, M. Malpetti, C. Le Draoulec, L. R. B. Spindler, A. O. G. Goodman, A. S. Lazar, R. A. Barker

**Affiliations:** 1 Department of Clinical Neurosciences, John Van Geest Centre for Brain Repair, University of Cambridge, Cambridge, UK; 2 Department of Clinical Neurosciences, Cambridge Centre for Frontotemporal Dementia and Related Disorders, University of Cambridge, Cambridge, UK; 3 Faculty of Medicine and Health Sciences, University of East Anglia, Norwich, UK; 4 Cambridge Stem Cell Institute, University of Cambridge, Cambridge, UK

**Keywords:** Dementia, Neurodegeneration, Huntington’s disease, Neurofilament light chain, Biomarker, Longitudinal

## Abstract

**Background:**

Growing evidence supports the value of neurofilament light (NfL) as a prognostic biomarker in premanifest Huntington’s disease (HD). To date, however, there has been no longitudinal study exceeding 3 years examining either its serial dynamics or predictive power in HD. We aimed to conduct the first such study.

**Methods:**

Serum NfL was sampled using ultrasensitive immunoassay at four timepoints across a 14-year period in a cohort of HD gene carriers (*n* = 21) and controls (*n* = 14). Gene carriers were premanifest at baseline. Clinical features of HD were evaluated by Unified Huntington’s Disease Rating Scale (UHDRS TMS), Montreal Cognitive Assessment (MoCA), Trail A/B task, Symbol Digit Modalities Task and semantic/phonemic fluency tasks.

**Results:**

14/21 HD gene carriers converted to prodromal or manifest disease by the final timepoint (“converters”). At baseline and each subsequent timepoint, NfL levels were higher in converters than in non-converters and controls (*p* = < 0.001–0.03, *η*_p_^2^ = 0.25–0.66). The estimated rate of change in NfL was higher in converters than in non-converters (*p* = 0.03) and controls (*p* = 0.001). Baseline NfL was able to discriminate converters from non-converters (area under curve = 1.000, *p* = 0.003). A higher rate of change in NfL was predictive of more severe motor (UHDRS-TMS *p* = 0.007, *β* = 0.711, *R*^2^ = 0.468) and cognitive deficits (MoCA *p* = 0.007, *β* = − 0.798, *R*^2^ = 0.604; Trail B, *p* = 0.007, *β* = 0.772, *R*^2^ = 0.567; phonemic fluency *p* = 0.035, *β* = − 0.632, *R*^2^ = 0.345).

**Conclusions:**

Our data suggest that (1) NfL longitudinal dynamics in premanifest/transitional HD are non-constant; rising faster in those closer to disease onset, and (2) NfL can identify individuals at risk of conversion to manifest disease and predict clinical trajectory, > 10 years from disease onset.

**Supplementary Information:**

The online version contains supplementary material available at 10.1007/s00415-024-12700-x.

## Introduction

Huntington’s disease (HD) is an autosomal dominant neurodegenerative condition caused by a CAG expansion repeat in exon 1 of the *huntingtin* gene, leading to an accumulation of mutant huntingtin protein (mhtt). It is characterised by a triad of psychiatric, cognitive and motor features. Clinical phenoconversion to manifest disease is currently defined by the emergence of unequivocal motor features, which typically occurs between ages 30 and 50. As such, HD exhibits a prolonged presymptomatic (“premanifest”) phase. Longer CAG repeat lengths predict a younger age of disease manifestation, but this leaves 30–50% of variance in onset age unaccounted for [1, 2]. Despite extensive efforts to identify additional metrics to enhance the prediction of onset [3–5], current methods remain limited. The enhancement of such prediction is important not only for prognostication for individual patients, but also for identification of study cohorts close to conversion, i.e. those most likely to benefit from disease-modifying agents currently in trial.

Growing evidence contends that neurofilament light protein (NfL) may constitute a biomarker able to provide such enhancement [6–8]. NfL is a protein subunit underlying the neuronal cytoskeleton and is consequently released following neuronal damage [9]. It has been found to be elevated during the presymptomatic period and to bear predictive power in a number of neurodegenerative conditions, for example Alzheimer’s disease [10–12], familial amyotrophic lateral sclerosis [13] and fronto-temporal dementia [14].

In HD, NfL has been found to discriminate disease stage cross-sectionally with a high degree of granularity, including during the premanifest phase [15, 16], and to correlate with a number of validated clinical and imaging measures of disease severity [15, 17, 18]. Indeed, NfL has been found to be superior to mhtt and other putative fluid biomarkers in these regards [17]. Cross-sectional studies have suggested a sigmoidal trajectory; becoming elevated versus controls as much as 17–24 years prior to predicted diagnosis [16, 19, 20], then increasing most rapidly during the transitional phase, before plateauing in advanced disease [6, 20, 21]. In support of this, multiple studies have found strong negative correlations between NfL levels and predicted years to onset of manifest HD; in some cases, exponential [18, 19, 21, 22]. Several studies have also found a baseline measurement of NfL to hold predictive power for subsequent clinical progression [15, 16, 20] and to be able to discriminate those at risk of converting to manifest disease within 3 years [20].

To date, however, there have been only three studies of longitudinal changes in NfL in HD [6, 16, 20], all of which were limited to a period of 2–3 years. There has also been no study in HD with clinical follow up > 3 years after a baseline blood sample. Indeed, to our knowledge, the longest serial blood NfL study in any established neurodegenerative condition bar multiple sclerosis has had a median follow up of 6.1 years [23] (a study in Alzheimer’s) and the longest period of clinical follow up following baseline NfL is 10 years, in a study of progressive supranuclear palsy [24].

Here we sought to address these knowledge gaps by reporting serial serum NfL versus clinical trajectories in a cohort of 14 controls and 21 premanifest HD gene carriers, studied at four timepoints across a 14-year period.

## Methods

### Study design and participants

We undertook retrospective analysis of data from a subset of participants enrolled in the Cambridge Huntington’s Sleep Study [25]; a longitudinal study in which participants underwent actigraphy, polysomnography and blood sampling at 4 timepoints between 2009 and 2023 (baseline: 2009–10, time 2: 2011–12, time 3: 2013–14, time 4: 2022–23). Approximately 50% of controls constituted healthy partners of recruited gene carriers; the remainder were recruited by local advertisement. Participants underwent assessment for motor features of HD at each timepoint, as well as cognitive assessment at baseline and Time 4.

Participants were enrolled to the original study based on the following criteria: (1) CAG repeat length ≥ 38 (in gene carriers) (2) premanifest disease status at baseline (in gene carriers) defined as Unified Huntington’s Disease Rating Scale Diagnostic Confidence Level (DCL) of 0–1 (equating to < 50% confidence of HD motor features) (3) no neurodegenerative disease (other than HD in gene carriers).

Participants were retrospectively enrolled to the NfL substudy based on the following further criteria: (1) banked excess serum samples at > 1 timepoint (2) age < 65 at baseline (3) no diagnosis of traumatic brain injury, renal impairment, neuroinflammatory or neurodegenerative disease bar HD during the study period (4) time 4 HD disease status (premanifest/prodromal/manifest) confirmed (in gene carriers). The age criterion was implemented due to the fact that, in healthy individuals, serum NfL levels demonstrate a slow linear increase of approximately 2%/year up to the age of 65, after which levels rise more rapidly and become increasingly variable between individuals [26, 27]. The renal impairment criterion was incorporated as serum NfL is renally cleared, such that renal impairment can lead to falsely elevated measurements [28, 29].

These criteria generated a cohort of 21 HD gene carriers and 14 controls. Ethical approval was granted by local ethics committees in accordance with the 1964 Declaration of Helsinki and all participants provided written informed consent (REC 03/187 and 15/EE/0445).

### Clinical assessment

Clinical features of Huntington’s disease were assessed using the following standard validated scales.

At each timepoint, a Unified Huntington’s Disease Rating Scale (UHDRS) [30] was undertaken, comprising:

- Total motor score (TMS): 0–124, higher score = greater deficit.
- Total functional capacity (TFC): 0 = 13; 11–13 = early stage; 7–10 = early-mid stage HD [31].
- DCL: 0–4, score ≥ 2 indicative of > 50% confidence of signs of manifest HD [31].

Cognitive assessment at baseline and Time 4 comprised:

- Montreal Cognitive Assessment (MoCA): test of global cognition, score 0–30, higher score = better performance [32].
- Trails test A: test of attention and psychomotor speed, timed 0–180 s, shorter time = better performance [33].
- Trails test B: test of executive function, timed 0–180 s, shorter time = better performance [33].
- Symbol Digit Modalities Test (SDMT): test of attention and psychomotor speed, score 0–110, higher score = better performance [34].
- Semantic and phonemic fluency task: items named in 60 s, fewer items = greater deficit [35, 36]

All motor assessments were undertaken by certified clinicians blinded to NfL results. Gene carriers were classed as prodromal where they had a UHDRS TMS ≥ 4 and a DCL ≥ 2, and manifest where they had a UHDRS ≥ 5 and DCL = 4. Years to predicted disease onset from baseline were calculated using the Langbehn equation [37] applied to age at baseline at 60% probability of symptom onset.

### Meso Scale Discovery assay

Extracted serum from venous blood samples was stored at -80 °C until processing after Time 4. NfL concentrations were determined using the Meso Scale Discovery S-PLEX Neurology Panel 1 (Human) kit according to manufacturer’s instructions, with an independent interplate control repeated across plates. All samples and standards were measured in duplicate. Plates were coated on the day of analysis and analysed using the Meso Sector 2400 Imager. Values were standardised to the independent interplate control with the lowest coefficient of variation (CV) (< 2%). Samples were re-rerun where CV exceeded the manufacturer’s recommended threshold (25%). Mean (SD) CV of final results was 10.62% (± 10.64). All values fell within the dynamic range of the assay (1.7–1400 pg/ml).

### Statistical analysis

Statistical analysis was undertaken in SPSS version 28.0 (IBM, Armonk, NY, USA), Prism version 9.5.1 (GraphPad Software, La Jolla, CA, USA) and R version 4.3.0 (R Core Team 2023) with R studio (RStudio team 2023, RStudio, PBC, Boston, MA). The threshold for statistical significance for all analyses was *p* < 0.05.

The presence of outliers was assessed for all variables at each timepoint according to disease group and was defined as values exceeding three standard deviations from the mean [38]. Where parametric tests were employed, data was transformed to a normal distribution via log transformation where necessary. The distribution of missing data between groups was assessed by Pearson *χ*^2^ for each variable.

Cross-sectional analysis of NfL concentrations was conducted by one-way ANOVA with post-hoc Tukey testing. To provide additional interrogation of our results, we mitigated against the possible effects of sample size by then also repeating analysis with bootstrapping (5000 replicates).

Longitudinal data from converters was explored using both linear and exponential models, with the former demonstrating a superior fit with our data (Supplementary Figure S1). Though both models demonstrated similar representational fits, applying an exponential regression to our data yielded parameters that generate an essentially linear model, indicating that our data were most accurately represented by linear parameters. Longitudinal changes in NfL were consequently analysed by linear mixed model, with group and time included as fixed effects, and individual participant intercepts and slopes incorporated as random effects. Baseline was adjusted to reflect the timing of the first available NfL sample for each participant. Age, sex, CAG repeat length, and body mass index (BMI) were added as fixed effects (covariates) and retained within the final model where their main effect met significance threshold. The inclusion of BMI reflects its influence on serum NfL levels as a function of total blood volume [28, 29]. Group*time interactions were used to test for group effects on longitudinal NfL changes. Mixed models were selected in place of repeated measures models to mitigate against the effects of missing data.

Annualised rate of change was calculated by taking the difference between Time 4 and Baseline or Time 1 values and converting to annualised rates according to the intervening time period. In all cases, the longest available period was used.

Predictive relationships were assessed by linear regression. Receiver operating characteristic (ROC) curve and area under the curve analyses were employed to investigate the ability of NfL to discriminate the clinical conversion of HD gene carriers. Cut off scores generated by these analyses were then used to undertake exploratory Kaplan Meier survival curve and Cox regression analyses.

For all ANOVA and regressions (including Cox regressions), age, sex, CAG repeat length and BMI were added as covariates using backward elimination. As per similar studies from our group, this represented the most stringent and meaningful method in the context [39], given the necessarily limited number of observations (given the rarity of the disease and highly extended time-period of follow-up) alongside a likelihood of combinatorial effects of covariates. This was deemed more stringent than covariate selection based on univariate analyses of our data, which would have led to the incorporation of one covariate only. Incorporated covariates were assessed for collinearity.

Due to the assessment of multiple clinical scales, final *p*-values for all regressions were adjusted for multiple comparisons via Benjamini–Hochberg correction (false discovery rate set at 0.05).

## Results

**Scheme 1:**
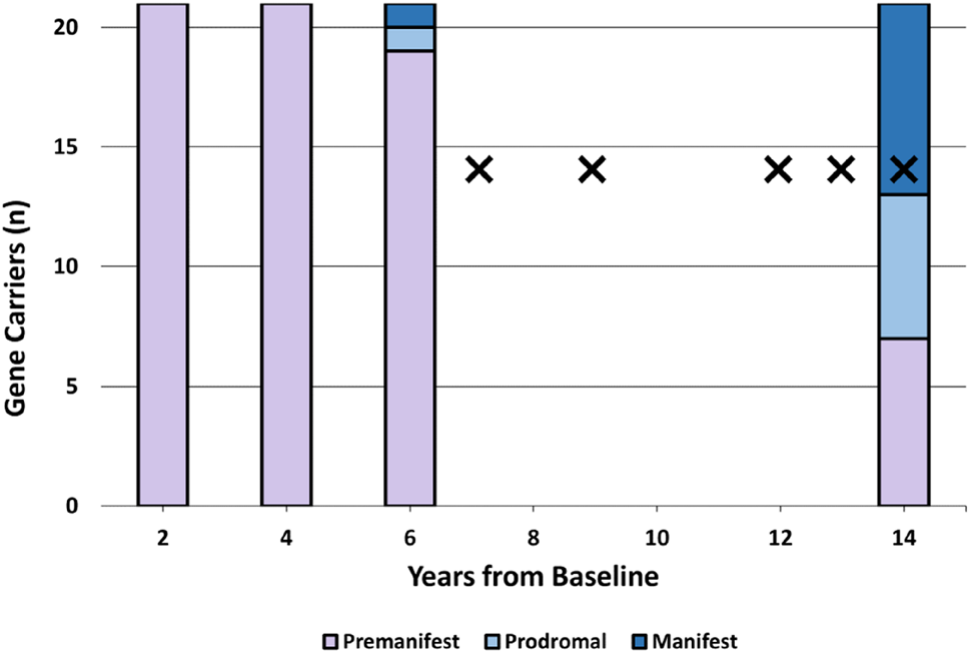
Clinical conversion trajectory of gene carriers. X denotes timing of conversion to manifest disease, where conversion could be confirmed to within 12 months (*n* = 5/8)

21 HD gene carriers and 14 controls participated in the study. All gene carriers were premanifest at baseline (2009–10) and Time 2 (2011–12). By Time 3 (2013–14), 1 gene carrier had converted to prodromal HD, and another had converted to manifest HD. Between Time 3 and Time 4, a further 12 gene carriers converted to prodromal (*n* = 5) or manifest (*n* = 7) HD. As such, 7/21 gene carriers remained premanifest by study completion (“non-converters”), whereas 14/21 had converted to prodromal or manifest HD (“converters”). The time of conversion to manifest disease could be confirmed to within 12 months in 5/8 manifest gene carriers; the mean ± SD years to conversion from study initiation within these individuals was 10.0 ± 2.9 years. The mean ± SD TFC of manifest individuals was 9.4 ± 2.7. Together, this indicates that our converter cohort predominantly reflected individuals in the prodromal or early stages of HD by study completion. Clinical trajectories are depicted in Scheme 1.

There was no statistical difference between converters, non-converters and controls with respect to potentially confounding factors, bar, as would be expected, a lower time interval in years to predicted onset from baseline among converters (Table 1). 5 gene carriers and 5 controls withdrew from blood sampling and cognitive testing at Time 4. These individuals were matched to participants remaining in the study on all relevant confounding factors apart from in a marginal reduction in years of education, and significantly lower predicted years to onset (Table 1). This latter finding reflects that all five gene carrier withdrawals were manifest of HD at Time 4.

**Table 1:** Cohort demographics

	Controls	Non-converters	Converters	p	Time 4 withdrawals	Time 4 non-withdrawals	*p*
*N*	14	7	14	N/A	10	25	N/A
Age at baseline	40.25 ± 13.53 (24.9–64.5)	38.56 ± 10.42 (24.3–51.0)	46.45 ± 9.21 (26.8–58.8)	ns	40.07 ± 9.77 (24.3–64.5)	43.32 ± 12.24 (28.4–58.8)	ns
% age > 65 at Time 4	33.33	0.00*	28.57*	ns	24.00	10.00	ns
% male	42.86	57.14	27.27	ns	20.00	44.00	ns
CAG repeat length	N/A N/A	40.14 ± 1.57 (38–43)	41.57 ± 2.28 (49–46)	ns	42.60 ± 1.67 (40–44)	40.63 ± 2.09 (38–46)	ns
Predicted years to onset	N/A N/A	27.43 ± 13.02 (16.0–55.9)	13.91 ± 6.15 (6.0–30.1)	**0.003**	8.93 ± 1.93 (6.0–10.8)	21.28 ± 10.8 (12.2–55.9)	**< 0.001**
Body Mass Index	26.21 ± 4.71 (17.7–33.3)	28.75 ± 5.18 (20.6–37.8)	27.49 ± 6.84 (19.9–43.2)	ns	30.01 ± 6.14 (25.7–34.4)	27.22 ± 5.74 (17.7–43.2)	ns
Years of education	15.40 ± 3.95	15.93 ± 2.65	13.54 ± 1.63	ns	12.40 ± 1.67	*15.25* ± *2.94*	**0.032**

Bold indicates *p* values < 0.05

All values are expressed as mean ± SD and range other than where % is stipulated. Group differences were assessed by Chi square/Fisher’s exact test/independent Student’s *T* test/Mann–Whitney *U*/One-Way Analysis of Variance (ANOVA)/Kruskal–Wallis tests, as applicable. Withdrawal demographics calculated across entire study cohort other than in CAG or predicted years to onset, where values reflect those for gene carriers only

*Ns* non-significant, *N/A* not applicable

*A *χ*^2^ test comparing %age > 65 at time 4 of converters and non-converters yields a non-significant *p*-value: 0.26

In addition, some participants omitted a proportion of cognitive tests or some blood sampling timepoints. Precise rates of participation, together with summary statistics, of these variables are detailed in Fig. 2B and Supplementary Table S2. Considering all participants and timepoints, NfL data were available in 97/140 (69%) possible observations, and clinical scores were available in 365/462 (79%) possible observations. There was no significant difference in the proportion of missing data per group in any variable, bar in comparatively lower missing NfL data among non-converters at baseline (*p* = 0.025). No values in any variable met outlier criteria.

1. **NfL is significantly higher in prodromal/manifest HD than in premanifest HD, and is positively associated with clinical markers of disease severity**

First, we assessed for differences in NfL levels between groups after phenoconversion to prodromal/manifest disease, i.e. by assessing Time 4 data.

**Fig. 1:**
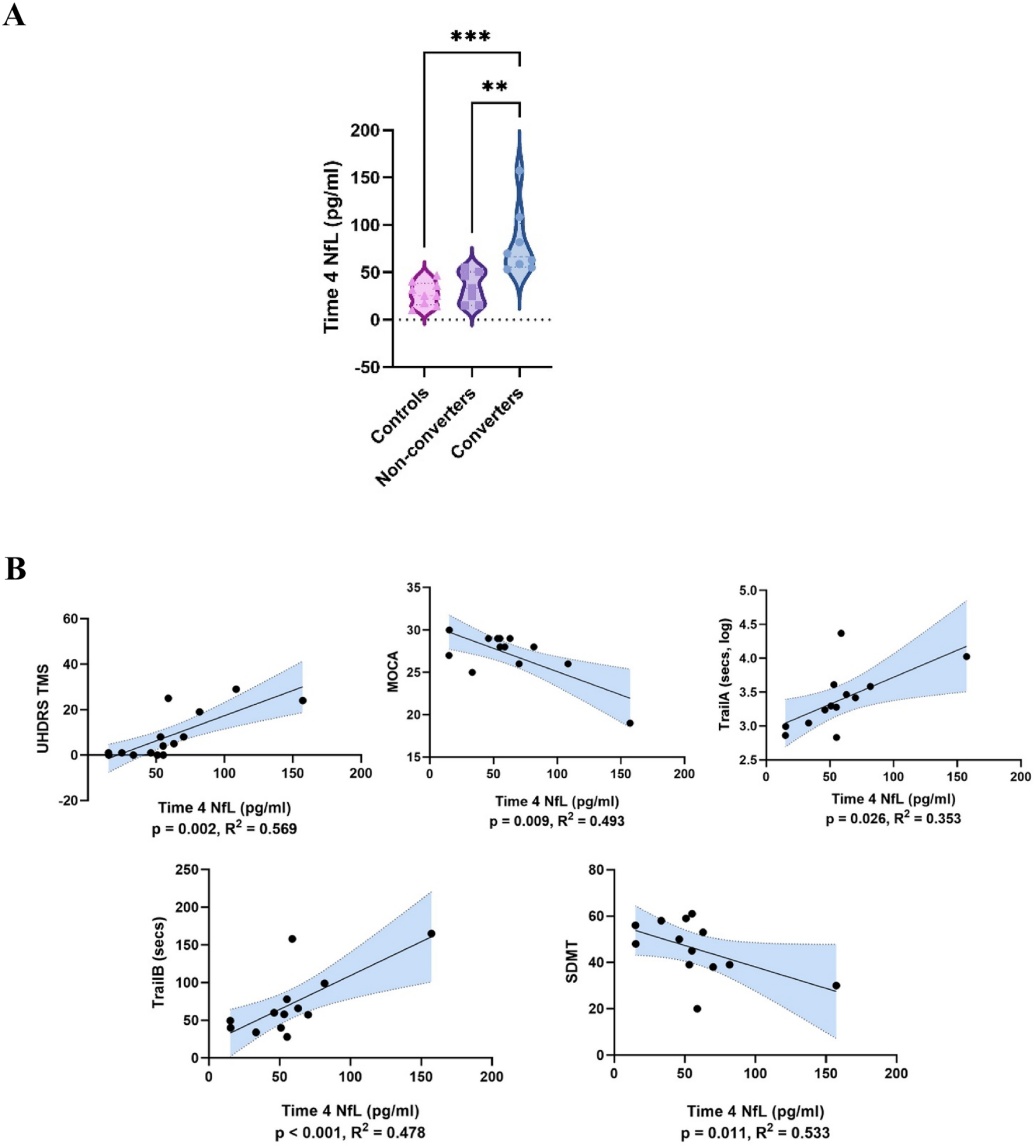
**A** Time 4 NfL concentrations. Group differences assessed by ANOVA adjusted for age, sex, CAG repeat length and BMI. ***p < 0.001; **p < 0.01; *p < 0.05. **B** Linear regression analyses of Time 4 NfL concentrations versus Time 4 clinical assessment scores among all gene carriers. Linear regressions adjusted for age, sex, CAG repeat length and BMI. *p* values corrected for multiple comparisons by false discovery rate (Benjamini–Hochberg correction). Shaded areas reflect 95% confidence interval. Results meeting significance threshold depicted

At Time 4, NfL concentrations were significantly higher in gene carriers who had converted to prodromal or manifest disease than in those who remained premanifest (*t* = 3.29, *p* = 0.006, Cohen’s *d* = 1.63) and controls (*t* = 4.02, *p* = < 0.001, Cohen’s *d* = 2.05) (ANOVA *η*_p_^2^ = 0.54). By contrast, NfL levels in non-converters did not significantly differ from controls (*t* = 0.920, *p* = 0.833) (Fig. 1A).

To provide additional confidence in our results, we mitigated against the possible effects of sample size by then also repeating analysis with bootstrapping. The results remained significant following this (Supplementary Table S3).

Higher Time 4 NfL levels among gene carriers were also associated with a more severe concurrent motor features (UHDRS TMS *R*^2^ = 0.569, *β* = 0.774, *p* = 0.002) and poorer scores on four cognitive scales (MoCA *R*^2^ = 0.493, *β* = − 0.732, *p* = 0.009; logTrail A *R*^2^ = 0.353, *β* = 0.638, *p* = 0.026; Trail B *R*^2^ = 0.478, *β* = 0.722, *p* = < 0.001; SDMT *R*^2^ = 0.533, *β* = − 0.726, *p* = 0.011) (Fig. 1B).

2. **NfL becomes elevated and rises more rapidly in the late premanifest/transitional phase of HD, compared to the early premanifest phase**

Next, we assessed for differences in NfL levels during the late premanifest/transitional phase of HD compared to the early premanifest phase and controls, by assessing for group differences cross-sectionally at Baseline, Time 2 and Time 3.

**Fig. 2:**
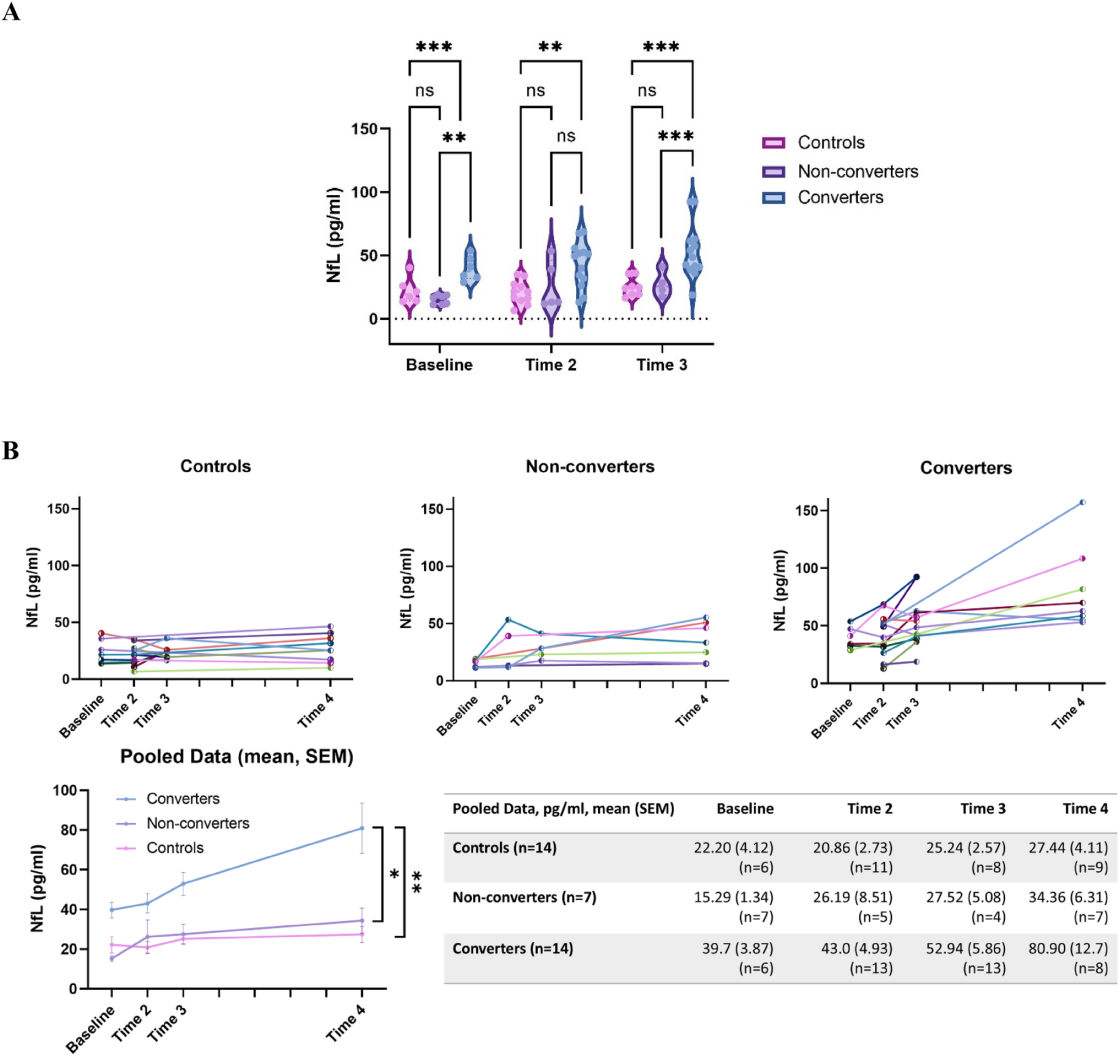
**A** Baseline, Time 2, and Time 3 NfL concentrations by group. Group differences assessed by ANOVA adjusted for age, sex, CAG repeat length and BMI. ***p < 0.001; **p < 0.01; *p < 0.05. **B** Longitudinal dynamics of NfL concentrations by group. Top = individual participants. Left shaded markers indicate sampling in first half of a given timepoint period, right shaded markers indicate sampling in second half of a given timepoint period. Bottom = pooled data. Error bars = SEM. **p < 0.01; *p < 0.05 in group*time interaction adjusted for age, sex, CAG repeat length and BMI

In cross-sectional analysis at each of these timepoints, NfL concentrations were higher in converters (i.e. those in the late premanifest/transitional phase) than in both non-converters (i.e. those in the early premanifest phase) and controls (*F* = 15.55, *p* = < 0.001, *η*_p_^2^ = 0.66; *F* = 4.05, *p* = 0.03, *η*_p_^2^ = 0.25; *F* = 5.56, *p* = 0.012, *η*_p_^2^ = 0.35 respectively). This reached statistical significance on post hoc pairwise analysis in all cases, bar between converters and non-converters at Time 2 (Fig. 2A). Findings again persisted following re-analysis incorporating bootstrapping (Supplementary Table S3).

We then assessed for group differences in the longitudinal dynamics of NfL across the study period.

Longitudinal patterns in NfL concentrations are depicted in Fig. 2B (to* p* = individual data; bottom = pooled data). Longitudinal modelling across the total study period by linear mixed model indicated significant main effects of both group (*F* = 8.79, *p* = 0.0094) and time (*F* = 17.6, *p* < 0.001), and a significant group*time interaction (*F* = 6.81, *p* = 0.006). Post-hoc pairwise comparison indicated that this interaction was significant with respect to both converters versus controls (estimate = 3.35 ± 0.92 (SEM), *p* = 0.001) and non-converters (estimate = 2.30 ± 1.00 (SEM), *p* = 0.033), suggesting a significantly faster rate of change in NfL among converters.

The mean (SD) annualised rate of change for the three groups generated from raw data was: controls 0.028 (± 0.47) pg/ml/year, non-converters 1.47 (± 1.24) pg/ml/year, converters 3.30 (± 3.13) pg/ml/year.

There was no significant difference between non-converters and controls in either cross-sectional (*p* = 0.161–0.707) or longitudinal modelling (estimate = 1.05 ± 0.99 (SEM), *p* = 0.304). This was also the case when longitudinal modelling was restricted to the first three timepoints (estimate = 3.20 ± 2.20 (SEM), *p* = 0.159).

Thus, together, these results suggest that NfL longitudinal dynamics are non-constant, increasing in parallel to healthy individuals during the premanifest phase far from onset, but becoming elevated and rising more rapidly during the late premanifest and transitional phase.

3. **NfL may identify individuals at risk of conversion to manifest disease and predict clinical trajectory over a subsequent 14-year period**

**Fig. 3:**
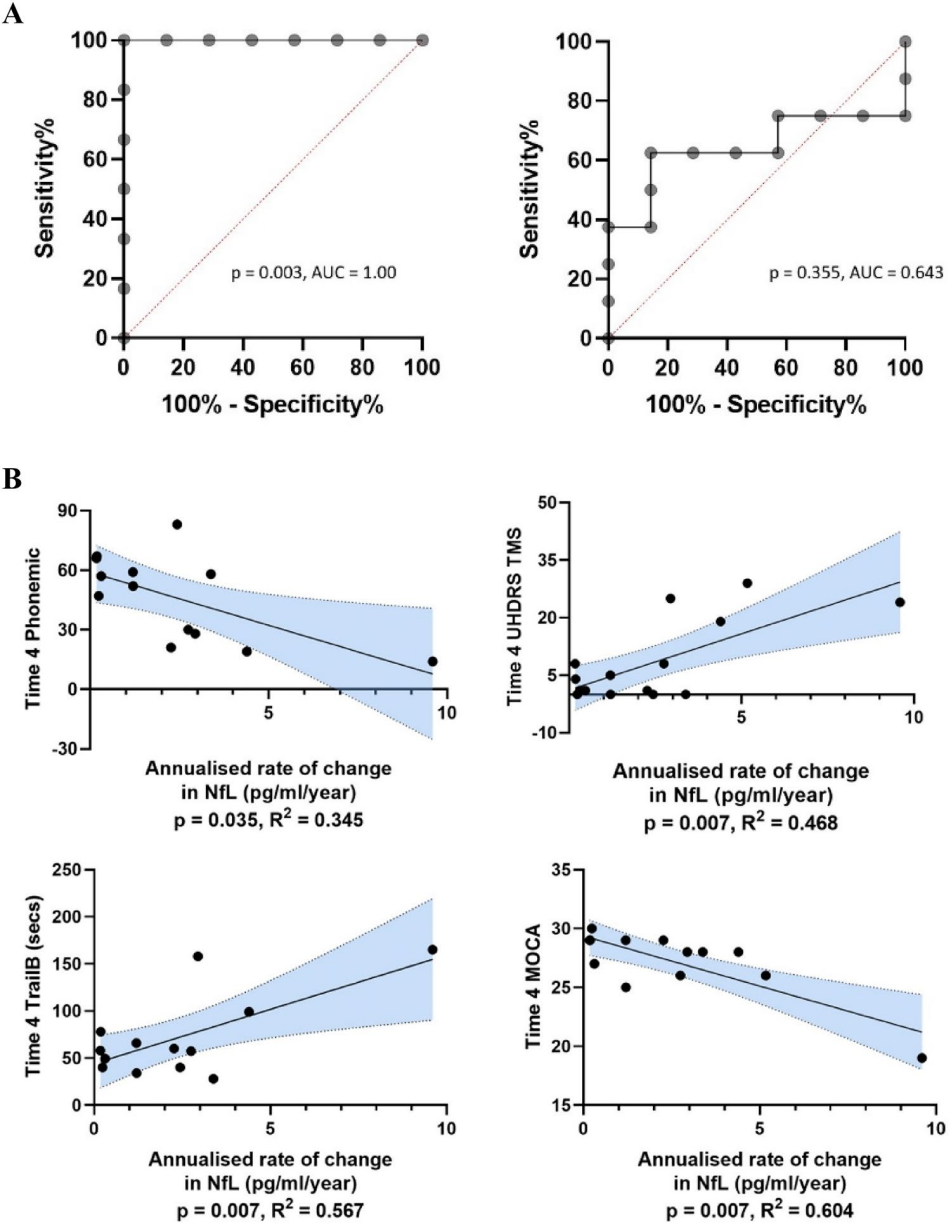
**A** ROC curve analysis of Baseline NfL concentrations (left) and annualised rate of change in NfL (right) versus discrimination of converter from non-converter gene carriers. *AUC* area under curve. **B** Linear regression analysis of annualised rate of change in NfL concentrations and Time 4 clinical assessment scores among all gene carriers. Linear regression adjusted for age, sex, CAG repeat length and BMI. *p* values corrected for multiple comparisons by false discovery rate (Benjamini–Hochberg correction). Shaded areas reflect 95% confidence interval. Results meeting significance threshold depicted

In ROC analysis, baseline NfL concentrations were highly effective in discriminating gene carriers who went on to convert during the study period from those who did not, exhibiting an area under curve of 1.000 (*p* = 0.003) (Fig. 3A). A cut off score of 24.06 pg/ml at baseline exhibited 100% sensitivity and specificity in determining this. A corresponding probability plot, depicting likelihood ratios of conversion within the study period for given baseline NfL concentrations, is provided in Fig. S4.

Annualised rate of change of NfL, however, did not exhibit parallel predictive utility: area under curve 0.643 (*p* = 0.355) (Fig. 3A). Nonetheless, annualised rate of change in NfL concentration in gene carriers was predictive of more severe motor features (UHDRS TMS *R*^2^ = 0.468, *β* = 0.711, *p* = 0.007) and poorer scores on three cognitive scales at Time 4 (MoCA *R*^2^ = 0.604, *β* = − 0.798, *p* = 0.007; Trail B *R*^2^ = 0.567, *β* = 0.772, *p* = 0.007; phonemic fluency *R*^2^ = 0.345, *β* = − 0.632, *p* = 0.035) (Fig. 3B).

We then used our NfL cut off score of 24.06 pg/ml to provide further exploratory analysis of the dataset. We used this cut off score to segregate gene carriers into high (*n* = 12) and low (*n* = 9) NfL groups according to NfL concentration at their initial sample. Longitudinal modelling of UHDRS TMS trajectories based on these groups demonstrated a significant group*time interaction (*p* = 0.047, Figure S5).

We also undertook Kaplan–Meier survival curve and Cox regression analysis based on these groups. This was necessarily exploratory, as the precise timing of conversion to manifest disease (accurate to within 12 months) could only be ascertained from clinical records for five of the eight participants who converted to manifest HD during the study. Limiting analysis to these five participants, versus those who remained premanifest throughout the study (*n* = 7), a log-rank (Mantel–Cox) test of the Kaplan–Meier survival curve showed a significant difference between the two groups (*χ*^2^(1) = 6.78, *p* = 0.009, Fig. S6). Cox regression, following adjustment for covariates, indicated that initial NfL > 24.06 pg/ml was a significant predictor of conversion to manifest HD within the study period (*p* = 0.028) with a HR of 12.43 (95% CI 1.73–250.38). The mean time to conversion in this group from initial NfL sampling was 9.0 ± 2.8 years.

## Discussion

Here we report the first exploration of the serial longitudinal dynamics and presymptomatic prognostic power of NfL in HD at a timeframe > 3 years. This novelty is particularly pertinent since extended longitudinal study is required before biomarkers can be considered for enrichment of current HD staging models (8). To our knowledge, this 14-year study represents the longest-duration study of serial blood NfL dynamics in any established neurodegenerative disease to date besides multiple sclerosis.

In HD gene carriers who remained premanifest during the 14-year study period, cross-sectional and longitudinal NfL dynamics did not differ significantly from that of healthy controls. By contrast, in gene carriers who converted to prodromal or manifest disease during the study period, NfL was elevated at baseline > 10 years from disease onset, and then rose more rapidly across time. This suggests that NfL dynamics in HD are non-constant, with faster increases in the late premanifest/transitional phase than in the early premanifest phase. This bears intriguing implications for the pathobiology of HD, suggesting that neuronal degeneration is non-constant; perhaps subject to a decompensation or feedforward process during the transitional phase.

From a clinical perspective, baseline NfL concentrations showed excellent ability to discriminate those who underwent symptomatic conversion to manifest/prodromal disease during the 14-year study period from those who did not, and the rate of change of NfL was predictive of a number of more severe motor and cognitive clinical outcomes.

The rarity of HD, together with the timeframe of the study, means this dataset represents a significant and rare window of insight into HD pathobiology despite its small size. Indeed, it is striking that we observed such marked group differences and associations despite this. Nonetheless, it is clearly imperative to acknowledge that this study is based on (1) a small cohort, and (2) with a proportion of missing data. We have mitigated against this through the use of comparative bootstrapping analysis, linear mixed models and analysis of distribution of missing data. However, results should be interpreted as exploratory and intended to inform a larger, statistically-powered validation study, rather than as absolute.

The study is also limited by the irregular spacing of timepoints, with a long interval between Time 3 and 4. Given that 12 of 14 converters transitioned between these timepoints, if an exponential NfL trajectory were indeed present, it is most likely to have occurred between these two timepoints. This therefore limits the precision with which the study can model specific longitudinal patterns. It is thus unsurprising that our data was better modelled by linear than exponential models, leading to our use of linear statistical approaches, but our estimates of rate of change are likely limited versus real-world data as a result and should also be viewed as exploratory.

Moreover, the results may also have been influenced by withdrawal bias, owing to the fact that all gene carriers who withdrew at Time 4 were converters. However, this would suggest that their inclusion is likely to only have further strengthened our results.

These limitations, as well as the influence of different assays, plasma versus serum NfL, cohort characteristics, and use of predicted versus actual years to conversion, mean that our results cannot be compared directly with those of other studies.

Nonetheless, our findings demonstrate a number of important parallels with existing studies. The observation that NfL is significantly elevated in prodromal/manifest versus premanifest HD cross-sectionally and is positively associated with clinical markers of disease severity, is in keeping with data from a number of prior studies [15, 17, 18].

Moreover, the non-constant trajectory of NfL modelled by our data corroborates the sigmoidal trajectory predicted by cross-sectional data and shorter-term longitudinal data [6, 20]. Indeed, it is notable that a similar pattern has been observed in a number of other neurodegenerative conditions, with steepest increases in NfL seen during transition to symptomatic disease, followed by a plateau during subsequent progression [11, 14]. This phenomenon is not yet fully explained but has been attributed to relative reduction in neuronal availability in advanced atrophy, and/or the development of autoantibodies to NfL [14].

Our finding of no difference between non-converters far from disease onset and controls is consistent with some studies [8, 40] but is, however, at odds with several others [16, 19, 20]. This may represent a consequence of our small sample size in comparison with these studies rather than a true absence of difference, especially given the unequal distribution of missing data between these groups at baseline.

Similarly, our finding that the annualised rate of change was a useful predictive marker of clinical outcome is at odds with findings of the three prior longitudinal papers [6, 16, 20]. This suggests that rate of change may only become useful once modelled over an extended period. In support of this possibility, NfL rate of change has been found to be a useful marker in AD [11]. Nonetheless, baseline NfL was superior to rate of change in our ROC analysis. The most meaningful application of NfL for predicting HD conversion and clinical progression may therefore transpire to be a combination of the two.

Our ROC analysis cut off score of 24.06 pg/ml is lower than that identified in parallel studies [20, 22], where a cut-off of approximately 45 pg/ml has been found to discriminate gene carriers far from onset from those close to phenoconversion. Once again, this likely reflects the comparatively expanded timeframe of our study. Nonetheless, the additional influence of other aforementioned cohort/methodological factors cannot be discounted.

It is possible that our results were influenced by a singular markedly elevated Time 4 NfL value, with a consequent corresponding prominently elevated annualised change rate (see Fig. 2B, 3B). However, this NfL value did not meet outlier criteria, was consistent upon re-assay, and was derived from the participant with the highest CAG repeat length in the cohort, providing a plausible biological basis [6, 20].

There is also the possibility that long-term storage may have influenced results, since samples were run as a batch on study completion rather than contemporaneously. However, this is mitigated by the fact that control samples were subject to the same storage conditions as those from patients, and it is notable that in a parallel study [16], storage over 3 years was not found to influence NfL assay performance.

Overall, this study extends current evidence that NfL may represent an important predictive biomarker in premanifest Huntington’s disease, and paves the way for a larger-scale validation study. The addition of an objective blood-based biomarker to current predictive scales would represent a major advance, since current estimates rely on i) clinical assessments, which may be variable or subjective, and ii) imaging markers, which are unattainable in many clinical and research settings [3–5]. Fortunately, such a study should soon be within reach, thanks to the advent of initiatives like Enroll-HD [41]. Given that some centres are beginning to incorporate NfL into clinical decision making in neurological disease [42], it is hoped that such work could eventually inform HD clinical care as well as clinical trials. We anticipate that both baseline and rate of change in NfL, added as enrichment markers to current staging models [5, 7, 8], are likely to provide best prognostic guidance.

## Supplementary Information

Below is the link to the electronic supplementary material.

Supplementary file1 (DOCX 398 KB)
