# Supplementary material for: A 14-year longitudinal study of neurofilament light chain dynamics in premanifest and transitional Huntington’s disease
Source: J Neurol. 2024 Oct 3;271(12):7572–82. doi: 10.1007/s00415-024-12700-x (PMC11588772; doi:10.1007/s00415-024-12700-x)
Supplement: Supplementary file 1 — Supplementary file1 (DOCX 398 KB) [file 415_2024_12700_MOESM1_ESM.docx]

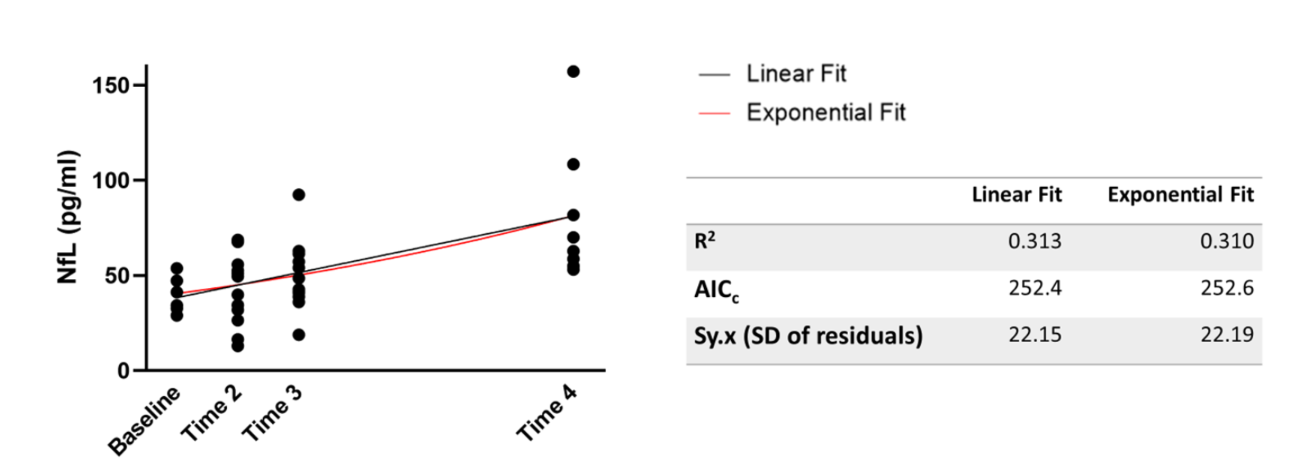


**Fig. S1. Linear and exponential modelling of longitudinal data from converters.** AIC_c_=Akaike Information Criterion corrected for small sample size. Sy.x (SD of residuals)=standard deviation of residuals

|  | **Baseline** |  |  |  | **TIME 4** |  |  |  |
| --- | --- | --- | --- | --- | --- | --- | --- | --- |
|  | ***Controls (n=14)*** | ***Non-converters (n=7)*** | ***Converters***  ***(n=14)*** | ***p*** | ***Controls (n=14)*** | ***Non-converters (n=7)*** | ***Converters (n=14)*** | ***p*** |
| **UHDRS TMS** | N/A | 0.14±0.38  (n=7) | 1.00±1.76  (n=12) | ns | N/A | 0.43±0.54  (n=7) | 17.93±10.67  (n=14) | **<0.001** |
| **MoCA** | 28.85±0.99  (n=13) | 28.71±0.95  (n=7) | 26.43±2.24  (n=14) | ns | 29.43±0.79  (n=7) | 27.80±1.92  (n=5) | 26.67±3.16  (n=9) | ns |
| **Semantic fluency** | 23.36±8.17  (n=14) | 23.00±6.97  (n=7) | 22.36±4.97  (n=14) | ns | 28.57±9.20  (n=7) | 25.67±11.84  (n=6) | 20.63±5.48  (n=8) | ns |
| **Phonemic fluency** | 48.71±13.35  (n=14) | 42.57±16.18  (n=7) | 43.93±19.33  (n=14) | ns | 55.57±9.98  (n=7) | 53.00±20.01  n=6) | 42.13±21.67  (n=8) | ns |
| **Trail A** | 29.29±7.43  (n=14) | 25.00±11.17  (n=7) | 32.64±8.86  (n=14) | ns | 25.29±5.78*  (n=7) | 21.33±4.12*  (n=6) | 44.75±18.51  (n=8) | **0.003** |
| **Trail B** | 49.21±20.34  (n=14) | 42.86±12.92  (n=7) | 54.21±15.18  (n=14) | ns | 49.43±13.26*  (n=7) | 41.92±11.39*  (n=6) | 99.54±43.16  (n=8) | **0.002** |
| **SDMT** | 50.00±9.40  (n=8) | 58.83±7.96*  (n=6) | 46.91±7.92  (n=11) | **0.035** | 54.43±14.58*  (n=7) | 55.33±5.20*  (n=6) | 38.63±10.07  (n=8) | **0.013** |

**Table S2.** **Clinical assessments by group at baseline and Time 4.** Group differences assessed by Student’s T-test/One Way ANOVA/Kruskal-Wallis as applicable and adjusted for age, sex and CAG repeat length. Values represent mean±SD. Ns=non significant. N/A=non applicable. *indicates p<0.05 on post-hoc Tukey testing versus converters.


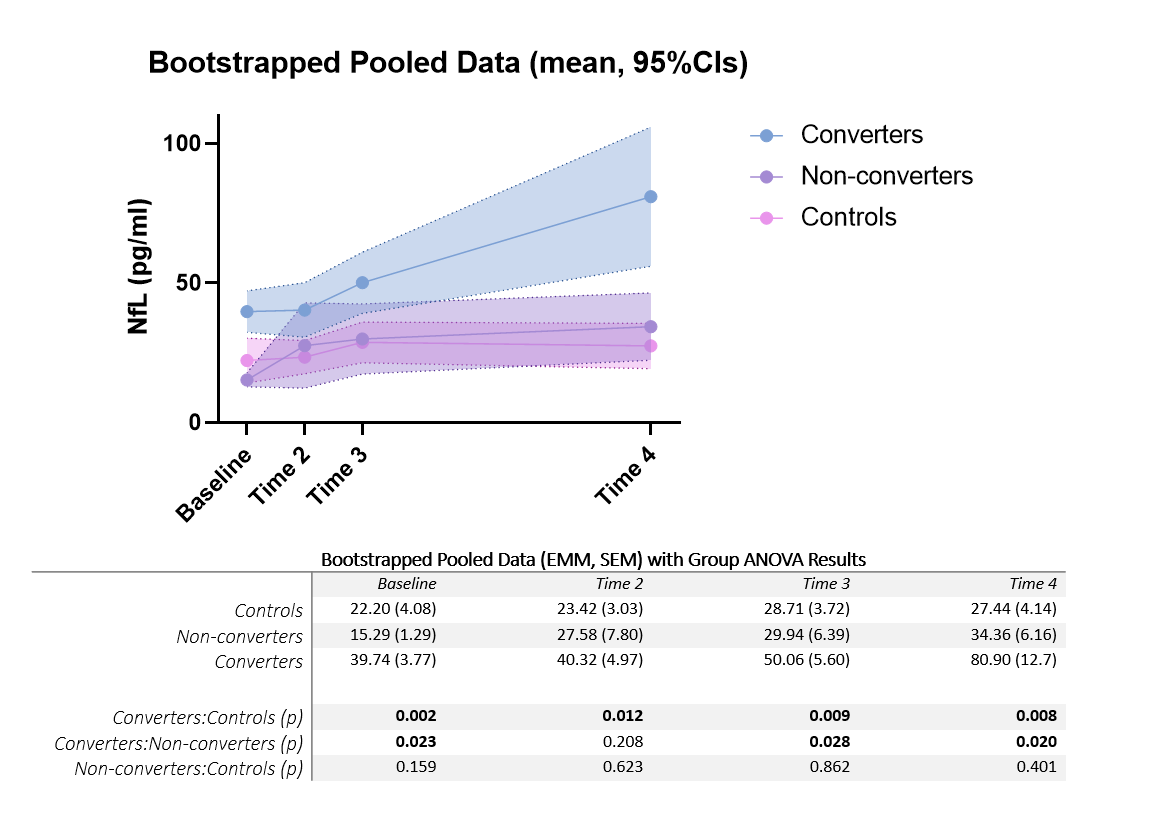


**Table S3. Estimated marginal means (EMM) of bootstrapped pooled data** plotted with 95% confidence intervals across all timepoints stratified by disease group. Accompanying table displays EMM and standard error of the means (SEM) values for each disease group at each timepoint as well as significance values for ANOVA post-hoc Tukey testing, adjusted for age, sex, CAG repeat length and BMI.


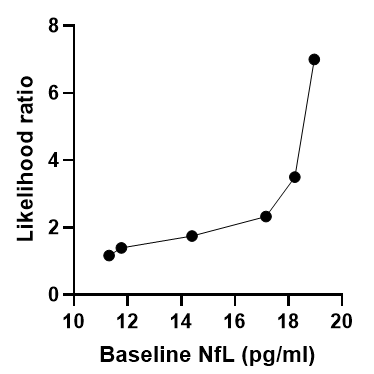


**Figure S4. Probability plot of conversion to prodromal/manifest HD within study period for given baseline NfL concentrations.**  Likelihood ratio represents sensitivity/1-specificity. The function becomes asymptotic and undefined at 24.06pg/ml, because at this value, the specificity and sensitivity are both 100%.


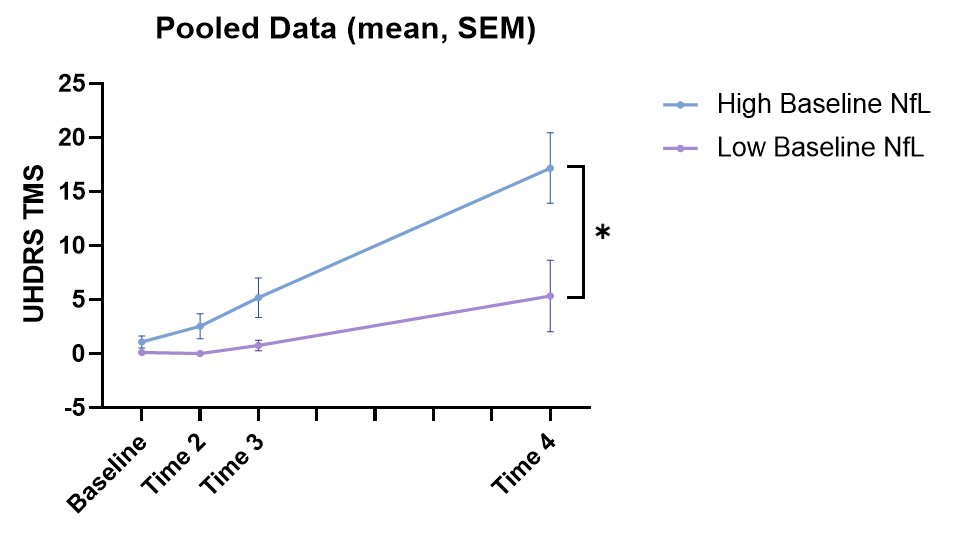


Initial NfL>24.06pg/ml

Initial NfL<24.06pg/ml

**Figure S5. Longitudinal changes in UHDRS total motor score according to initial NfL concentrations.**  *p<0.05 for group*time interaction adjusted for age, sex, CAG repeat length and BMI.


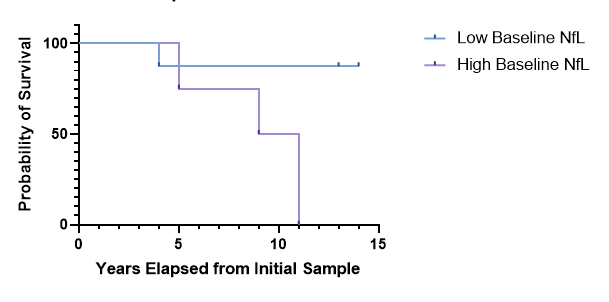


Initial NfL>24.06pg/ml

Initial NfL<24.06pg/ml

**Conversion free (%)**

**Log-rank p=0.009**

**Figure S6.**  **Kaplan Meier estimates of conversion to manifest HD across the study period, according to initial NfL concentrations**. Event defined as conversion to manifest HD. Estimates limited to gene carriers for whom time of conversion to manifest HD could be ascertained to within 12 months (n=5) versus participants who remained premanifest throughout the study period (n=7).
